# The impact of nanomaterials on autophagy across health and disease conditions

**DOI:** 10.1007/s00018-024-05199-y

**Published:** 2024-04-17

**Authors:** Ida Florance, Marco Cordani, Parya Pashootan, Mohammad Amin Moosavi, Ali Zarrabi, Natarajan Chandrasekaran

**Affiliations:** 1 Centre for Nanobiotechnology, Vellore Institute of Technology, Vellore, Tamil Nadu 632014 India; 2 Department of Biochemistry and Molecular Biology, Faculty of Biological Sciences, Complutense University of Madrid, 28040 Madrid, Spain; 3 Instituto de Investigaciones Sanitarias San Carlos (IdISSC), 28040 Madrid, Spain; 4 Department of Molecular Medicine, Institute of Medical Biotechnology, National Institute of Genetic Engineering and Biotechnology, P.O Box 14965/161, Tehran, Iran; 5 Department of Biomedical Engineering, Faculty of Engineering and Natural Sciences, Istinye University, Istanbul, 34396 Turkey; 6 Department of Research Analytics, Saveetha Dental College and Hospitals, Saveetha Institute of Medical and Technical Sciences, Saveetha University, Chennai, 600 077 India; 7 Graduate School of Biotechnology and Bioengineering, Yuan Ze University, Taoyuan, Taiwan

**Keywords:** Autophagic flux, Autophagy blockade, Environmental toxicity, Nanoplastics, Nanoparticles, Regulated cell death

## Abstract

Autophagy, a catabolic process integral to cellular homeostasis, is constitutively active under physiological and stress conditions. The role of autophagy as a cellular defense response becomes particularly evident upon exposure to nanomaterials (NMs), especially environmental nanoparticles (NPs) and nanoplastics (nPs). This has positioned autophagy modulation at the forefront of nanotechnology-based therapeutic interventions. While NMs can exploit autophagy to enhance therapeutic outcomes, they can also trigger it as a pro-survival response against NP-induced toxicity. Conversely, a heightened autophagy response may also lead to regulated cell death (RCD), in particular autophagic cell death, upon NP exposure. Thus, the relationship between NMs and autophagy exhibits a dual nature with therapeutic and environmental interventions. Recognizing and decoding these intricate patterns are essential for pioneering next-generation autophagy-regulating NMs. This review delves into the present-day therapeutic potential of autophagy-modulating NMs, shedding light on their status in clinical trials, intervention of autophagy in the therapeutic applications of NMs, discusses the potency of autophagy for application as early indicator of NM toxicity.

**Graphical Abstract:**

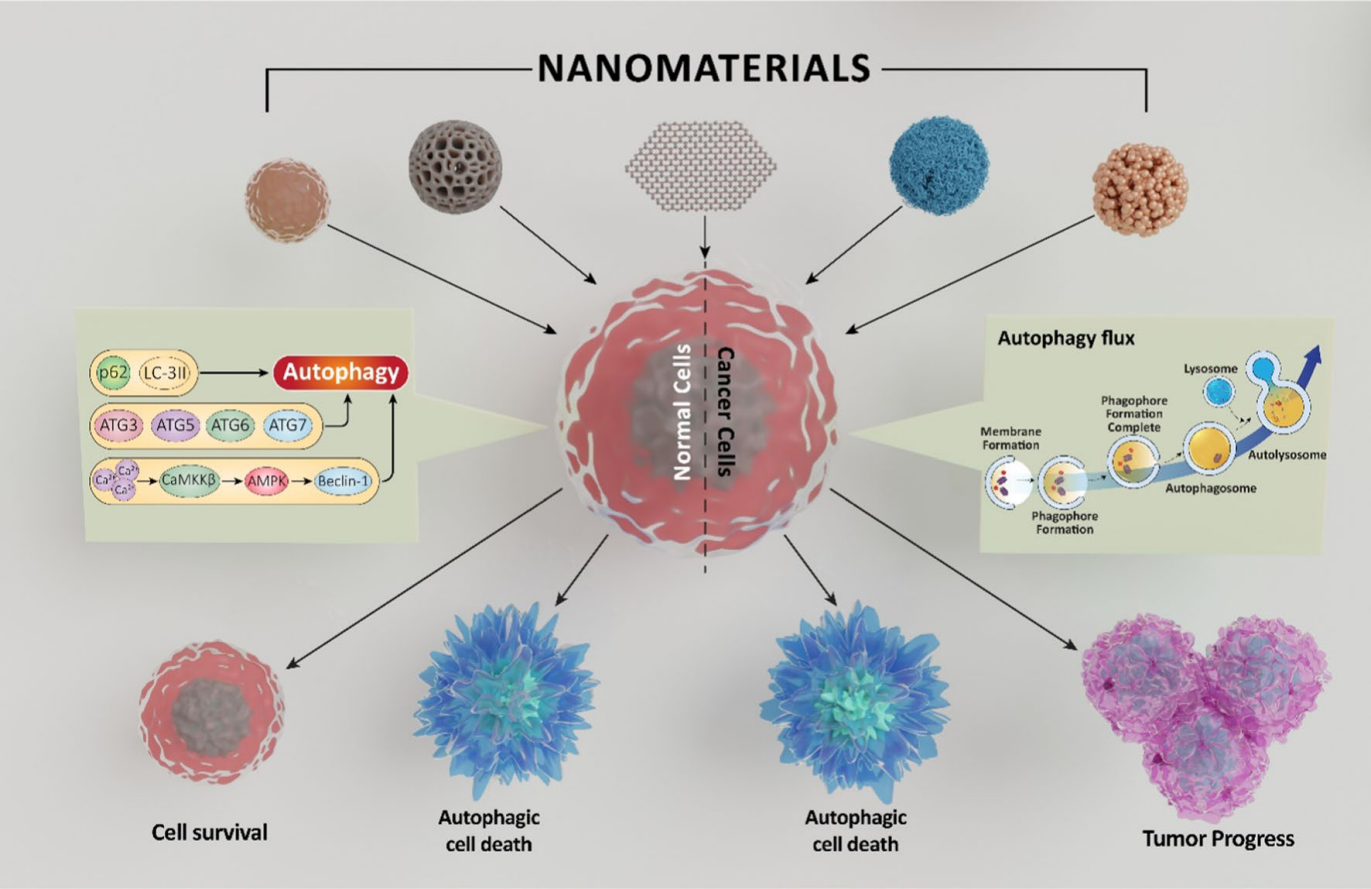

## Introduction

Autophagy is a naturally conserved self-degradative mechanism responsible for the removal of damaged, abnormal, and long-lived cellular biomacromolecules and organelles [1]. Under physiological conditions, autophagy occurs at a basal rate, to constitutively regulate intracellular recycling of cell components [2]. Based on the pattern of cargo delivery into lysosomes, three forms of autophagy, including microautophagy, chaperone-mediated autophagy (CMA), and macroautophagy, have been identified. In microautophagy, the target cellular contents are invaginated by the lysosomal membrane [3]. CMA is a highly specific type of autophagy that requires a KFERQ sequence in the target proteins. Proteins with the consensus sequence are identified by a chaperone protein called HSC70 and are sequestered to the lysosomal membrane [3]. The transport of cargo into the lysosomal lumen occurs via a lysosomal membrane receptor called LAMP-2A [4]. Macroautophagy, hereafter called autophagy, is a major type of autophagy predominantly used by the cells for the removal of cellular debris and damaged organelles [3]. This mechanism is initiated by the formation of unique double-membraned structures called autophagosomes that sequester and engulf target proteins called “cargo” and delivers them into lysosomes for degradation [5, 6].

This intricate process of cellular housekeeping, autophagy, sets the stage for understanding the impact of nanomaterials (NMs), as they interact with and influence these fundamental cellular activities. Due to unique physicochemical properties of nanoparticles (NPs), including high surface area, high functionality, easy penetrating into cells and, they have attracted significant attention to be used in industry and medical applications. There are several approaches to synthesizing NMs which can take on a variety of morphologies, such as disks, cubes, rods and spheres [7]. Because of their smaller size, NMs are highly reactive chemically and are easily prone to aggregation and agglomeration [8]. Thus, surface modification is required to prevent agglomeration and enable surface functionalization tailored to specific applications. [9].

NMs are broadly classified into organic and inorganic types, with carbon-based NMs counted as a separate class due to their wide range of spectroscopy proprieties. Examples of carbon-based NMs include Carbon Nano Tubes (CNTs), nanocomposites, nanofibers, nanowires, quantum dots and dendrimers [10, 11]. nPs, on the other hand, are NMs ubiquitously present in the environment and pose a major threat for both animal and human health. They can be synthesized for various applications or obtained from the fragmentation of larger plastics.

There are numerous reports on the role of NMs in modulating autophagy. NMs-induced autophagy had been considered both as mechanism of nanotoxicity and as a defense mechanism against nanomaterial-induced toxicity [12]. Distinct from previous reviews, this manuscript not only offers a comprehensive exploration of the interaction of NMs with autophagy across therapeutic and environmental contexts but also delves into the state-of-the-art knowledge about the importance of autophagy flux or blockade in NP studies. For the first time, we deeply discuss the pro-death roles of autophagic NPs and its connection to regulated cell death (RCD), and offer new therapeutic applications of autophagic NPs, as controllable autophagic tools or as carriers for autophagy-modulating drugs. Our review uniquely synthesizes these insights against the backdrop of recent significant reviews, providing a comprehensive view of the multifaceted roles of NMs in autophagy regulation. In this review, we discuss the contrasting roles of NMs in modulating autophagy in both therapeutic and environmental contexts, delving into the molecular mechanisms involved, potential health implications, and the broader significance of these interactions in advancing our understanding of NM applications in biomedicine and environmental health.

## Autophagy-regulating pathways

As a highly regulated cellular process, autophagy activation results from the integration of several signaling pathways. Mammalian target of rapamycin (mTOR) is a serine/threonine kinase that plays a crucial role in the regulation of cellular metabolism and growth [13]. It is also a key regulator of autophagy, serving as a negative regulator of the process. Under nutrient-rich conditions, mTOR complex 1 (mTORC1) is activated and promotes the inhibitory phosphorylation of ULK1 (unc-51 like autophagy activating kinase 1), a protein that plays a crucial role in the initiation of autophagy [14]. However, when nutrient levels are low, mTORC1 activity is inhibited, leading to activation of ULK1 and initiation of autophagy [15]. The importance of mTOR is extensively demonstrated that a number of pharmacological inhibitors have been developed to activate autophagy for therapeutic purposes in a number of morbidities [16].

**Fig. 1:**
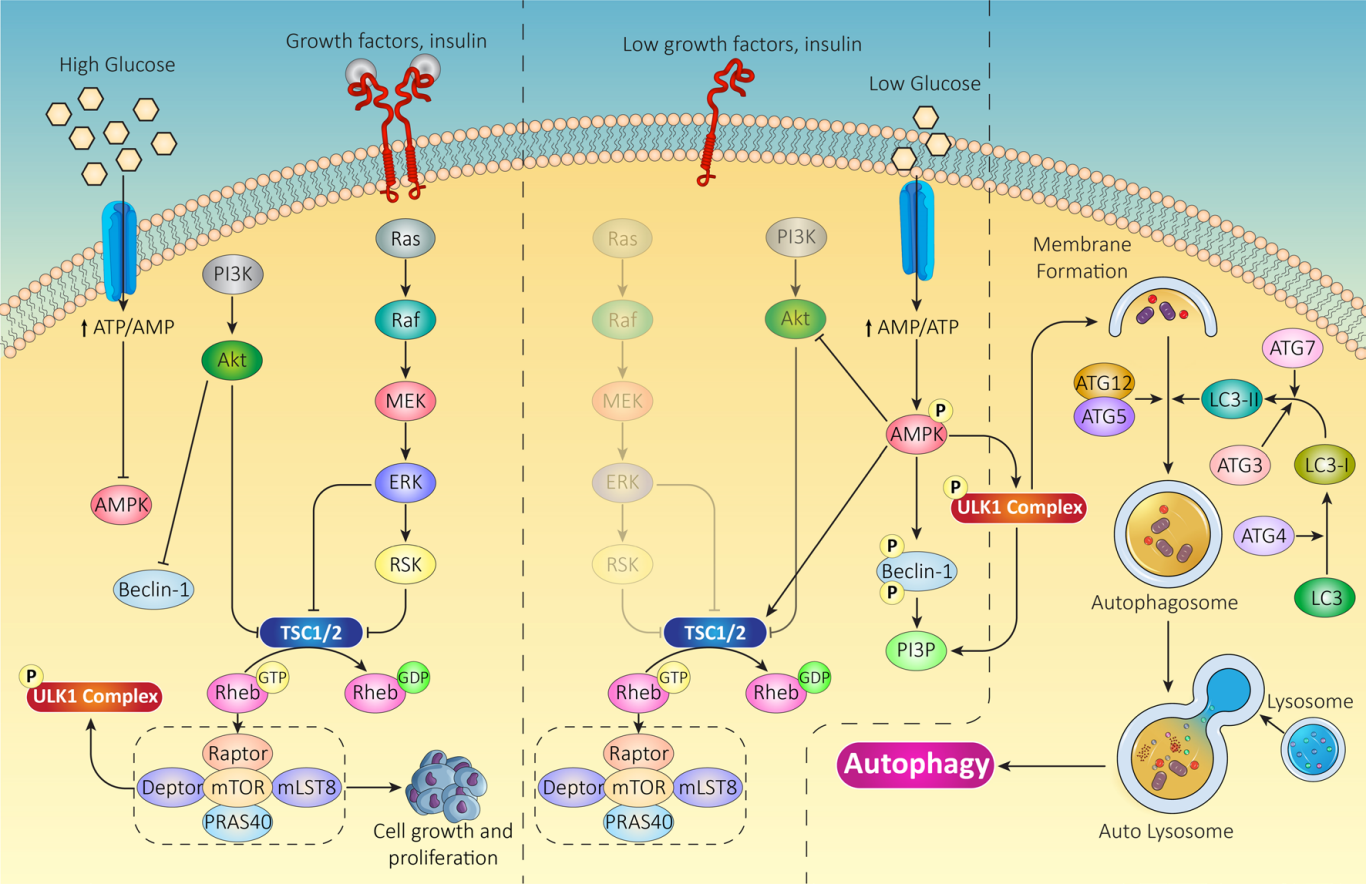
Autophagy and nutrient availability. Nutrients availability differentially regulates mTOR and AMPK signaling pathways which interact in a complex interplay

AMP-activated protein kinase (AMPK) is another master regulator of autophagy in response to low energy status in the cell [17]. In conditions of energy deprivation, AMPK is activated by the increased AMP/ATP ratio, which promotes the activation of autophagy to generate ATP via the recycling of cellular components. Notably, AMPK acts as antagonist to mTOR activity since it can directly phosphorylate and activate ULK1 complex, leading the formation of the isolation membrane, the first step in autophagosome biogenesis [14] AMPK also activates autophagy through the phosphorylation and inactivation of the mammalian class III phosphatidylinositol-3-kinase (PI3K) complex, which is a negative regulator of autophagy [18]. This inactivation leads to the dephosphorylation and activation of BECN1 (Beclin-1), a critical component of the autophagy initiation complex [19]. The main mechanisms regulating mTOR and AMPK axis are reported in Fig. 1.

Overall, both mTOR and AMPK processes play a critical role in coordinating cellular energy status and metabolism with autophagy to maintain cellular homeostasis. However, it is important to note that their role is complex and context-dependent, and its regulation of autophagy is also influenced by other signaling pathways and cellular conditions [20]. In this regard, autophagy can be activated by several physiological and pathological conditions. During physiological conditions like starvation and nutrient deprivation, cells tend to catabolize the damaged components thereby generating substrates for sustained ATP production [21]. While autophagy is activated during physiological stressful conditions such as starvation [22], hypoxia [23] 23 and infection [25] to sustain cell survival and homeostasis, pathological conditions like neurodegenerative diseases and cancers require inhibition of autophagy to overcome cytoprotective roles of autophagy and promote disease pathogenesis [26]. For instance, in Alzheimer's disease, impaired autophagy contributes to the accumulation of β-amyloid plaques and neurofibrillary tangles in the brain [27]. In muscular dystrophies, defective autophagy results in the accumulation of dysfunctional mitochondria and impaired muscle regeneration [28]. However, autophagy has a double-faced role during cancer. Whereas inhibition of autophagy activity is required to promote cancer formation by increasing oncogene-induced tumorigenesis and DNA damage accumulation, an increased level of autophagy maintains formed cancer cells survival and contributes to drug resistance [29, 30]. Therefore, autophagy can plays either pro-death or pro-survival roles in human diseases so that both its inhibition and activation may have therapeutic values for patients [31, 32].

Further, entry of foreign particles triggers autophagy which in response acts as a first line of defense [33]. Nevertheless, it is possible that the autophagy activated in response to the entry of foreign particles or by the foreign particles can be both pro-survival and pro-death [34]. However, this relies on the surface interactive properties of the particle. Contrastingly, foreign particles can also impair autophagy flux. Inhibited and impaired autophagy can be activated or enhanced using autophagy inducers. Similarly, autophagy inhibitors can be used to counteract over-activated autophagy. As mentioned previously, impaired autophagy is associated with several metabolic disorders and diseases [35, 36]. Consequently, drugs that target these conditions are being developed to induce autophagy, either directly or indirectly. Moreover, autophagy induction is also a direct therapeutic strategy for several disease conditions [37]. Phytocompounds, their synthetic analogs and chemical compounds, have been reported to modulate autophagy [38–41]. Notably, natural compounds have been shown to influence autophagy, with significant implications for the treatment of conditions such as stroke [42]. NMs, particles or constituents ranging from 1 to 100 nm (nm) in at least one dimension [10], have recently gained a lot of interest for their potential application in several fields, including for the treatment of cancer and other diseases [43, 44]. It has been reported that several NMs can modulate autophagy from synthesized NPs to nPs found in the environment [45, 46]. By identifying the ability of NMs to regulate autophagy, researchers may discover powerful modulators of this cellular process.

## Nanomaterials and their applications in therapeutics

Nanoscale materials possess unique properties and behavioural features including large surface area to volume ratio, and optical, electrical and/or magnetic properties which attribute to their medical applications, to prevent, diagnose, monitor and treat diseases when used directly or merged/added to a pre-existing product [47].

Therapeutic NPs are broadly classified into two types: nanocrystalline and nanostructured. Nanostructured materials are further classified into lipid-based, polymeric and non-polymeric structures [48]. Nanogels, micelles, nanoparticles, drug conjugates, protein nanoparticles and dendrimers are examples of polymeric NMs [49, 50] Similarly, quantum dots, metallic nanoparticles, carbon nanotubes, silica-based particles and nanodiamonds are examples of non-polymeric structures [51]. Solid-lipid nanoparticles, nanostructured lipid carriers and liposomes are categorized under lipid-based NMs [52, 53]. Therapeutic agents that are crystalline in nature are used in combination to form nanocrystalline particles and are used for several clinical applications [50]. Grapefruit-derived nanovectors (GNVs) are nanoparticles synthesized from grapefruit-derived lipids. They are developed to transport chemotherapeutic agents like siRNA, DNA and other proteins to different cells [54]. Further, hyaluronic acid-chitosan based nanocomposite hydrogels had been developed for photothermal chemotherapy against cancers [55].

Inorganic nanoparticles like Zinc oxide (ZnO NPs), silver nanoparticles (Ag NPs) and gold nanoparticles (Au NPs) are majorly exploited for treatment of cancers and anti-angiogenic effects. For example, modified AuNPs have been recently employed to overcome pancreatic cancer chemoresistance to Gemcitabine [56]. Similarly, Ag NPs and ZnO NPs have been extensively reported for their antibacterial activity [57, 58]. Additionally, fullerene derived NPs were reported to have free radical scavenging activity [59]. In wound healing, nanoparticles with anti-bactericidal properties are desirable. In such cases, nanoparticles like Ag NPs, Cu NPs and ZnO NPs are predominantly used for their anti-microbial and anti-bactericidal properties [60]. Recently, self-powered and implantable ZnO nanowire-based electronic skin had been developed to monitor skin health transdermal [61]. Electrospun nanofibers are effectively used in wound dressing as they regulate wound humidity [62]. Further, carbon dots were considered as potential nanostructures to target RNA (nucleic acid) and capsid proteins of SARS‐CoV‐2 using photodynamic therapy [63]. In addition, carbon nanotubes have been employed in drug delivery, treatment of neurodegenerative diseases, tissue regeneration, infection therapy, DNA delivery for gene therapy and anti-tumor immunotherapy [64].

Interestingly, nPs have also been used as therapeutic agents. For example, sodium polystyrene sulfonate remains the current treatment option to decrease serum potassium levels and clinically manage hyperkalemia [65]. Besides being therapeutic agents by themselves, NMs are mostly known for their application as careers of drugs and nano-vehicles and vectors [66].

While NMs have shown immense promise in a range of therapeutic applications, from targeted drug delivery to novel treatments for various diseases, they also present new avenues to address existing challenges in pharmacology. Although many anti-cancer and anti-tumor drugs that are hydrophobic, they face limitations in clinical applications due to their solubility and metabolism in biological systems. Recent advancements in nanotechnology have improved nano-drug delivery systems that can overcome such challenges including non-targeted cytotoxicity [67]. However, some anti-tumor drugs can induce mild autophagy, which may promote tumor cell survival, and are prone to rapid clearance by macrophages. Nanocomposites can increase drug accumulation in tumor cells and prevent multi-drug resistance [68], but overcoming this resistance through this approach remains a challenge.

## Environmental and health concerns of nanomaterials

NMs have become an integral part of our daily lives, and exposure to them has become unavoidable. The unceasing use of synthetic nanoparticles has resulted in their increased release into environmental media such as air, water, and soil [69]. NMs can be released into the environment during the production of nano-based raw materials, the use of products made of NMs, and during the disposal of such products [70].

The form of materials encountered by humans and the environment remains a decisive factor while assessing the environmental risks associated with NMs. Similarly, the behavior of NMs released into the environment is a major concern. Additionally, environmental risks associated with NMs are also linked to their properties, such as stability, shelf-life, solubility in water and body fluids, ability to agglomerate, tendency to interact with other nanoparticles, chemicals, and surfaces. Owing to their active surfaces, NMs can mobilize pollutants and pose a significant threat to groundwater. Similarly, their smaller size can lead to distribution in the air [71, 72].

In contrast, highly stable NMs can remain unaffected in the environment and ultimately reach biological systems, accumulating there [73]. Furthermore, NMs released into the environment can undergo surface modification caused by several environmental factors. However, it is unclear to what extent changes in morphology and surface properties can affect the toxicological properties of NMs. For example, polystyrene NPs were reported to impair lipid metabolism in macrophages without having a direct impact on the viability of cells [74]. NMs made of basic materials that are soluble tend to lose their nanostructure post entry into biological fluids [75, 76]. NMs undergoing changes in kinetics could also exert nano-specific toxicity [77].

## Autophagic interventions in the uptake of NPs, NMs and nPs

Autophagy plays a vital role in cellular responses against NMs and nPs exposure [78]. Whether it promotes survival or death largely depends on the type of NP or nP and the severity and timing of exposure [12]. While some NMs might be seen as foreign entities by cells, others, especially those supported by intracellular macromolecules, can be challenging for cells to recognize and process [12]. Activation of autophagy is a major cellular response against NP and nP entry [79]. For NMs that have entered the cell, the endo-lysosomal pathway tries to clear them [80]. However, nPs can also accumulate within autophagosomes, indicating the key role of autophagy in their clearance [79]. On the therapeutic side, this can pose a challenge, as autophagosomes might engulf NMs before they exert their therapeutic effects, making conditions that compromise autophagy more favorable for therapeutic outcomes [81].

Micro and nanoplastics enter biological systems through ingestion, inhalation, and skin contact [82]. NMs mainly enter cells via endocytosis, with other non-endocytic pathways also playing a role [83, 84] Autophagy can impact this uptake both directly and indirectly. For instance, when nPs are coated with serum proteins from FBS, autophagy can be compromised in macrophages [85]. Conversely, when autophagy is inhibited, there can be an enhancement in nPs uptake through phagocytosis [86]. While nanotechnology offers promising therapeutic interventions, our ecosystem faces contamination from micro and nanoplastics. Understanding autophagy as a cellular response to nanomaterial exposure and as a potential mechanism for nanomaterial-based therapies is crucial. Bridging the gap between environmental NMs and therapeutic ones requires a deeper exploration of autophagy. Continued research into the effects of nanoscale and submicron plastics on cellular processes and internal organs is vital, as is the development of NMs that can efficiently modulate the autophagy pathway for future treatments and clinical trials.

## Autophagy modulating effects of NPs: autophagy flux or blockade

### Nanomaterials as inducers of autophagy flux

**Fig. 2:**
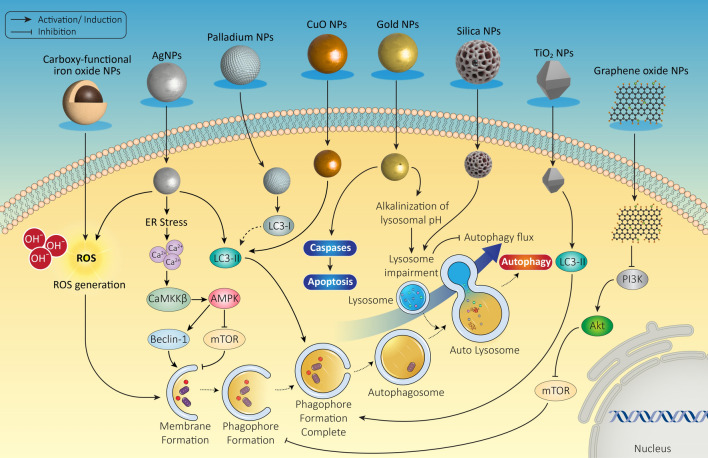
Schematic representation of potential modulatory effects of NMs on autophagy pathway

Induction or blockade of autophagy flux is defined by the increase and decrease in autophagic degradation activity [87]. NPs have an intrinsic ability to regulate the autophagy pathway at various stages, making them potential candidates for inducing autophagy (Fig. 2). In polymeric NPs, such as PLGA NPs, which are taken up and degraded by lysosomes, an increase of acidification in lysosomes is occurred, leading to the induction of autophagic flux along with a decrease of SQSTM1/p62 [88, 89].

A wide variety of NPs, including QDs, alumina NPs, zinc oxide NPs, Au NPs 22 nm, silica NPs, TiO_2_ NPs, CNTs and ultra-small super paramagnetic iron oxide (USPION) NPs, also induce autophagy flux at least in part by the inhibition of MTOR signaling pathway or by inducing the expression and/or phosphorylation of autophagy-related and BCL2-family proteins involved in autophagy [90, 91]. ROS production by NPs may also play a role in autophagy induction [92].

Fullerenes, CuO, TiO_2_, Nitrogen-doped TiO_2_, cerium dioxide, iron oxide and neodymium(III) oxide (Nd2O3) NPs increase autophagy flux via ROS production or other mechanisms that exerting significant cytotoxic effect on breast, cervix and lung cancer cells [93, 94]. In addition, photoactivation of graphene QDs and N-TiO2 NPs can induce consolable autophagy flux in cancer cells [95, 96]. The autophagic effects of NPs are dependent on their size and physico-chemical characteristics. For example, CdSe QDs with a size larger than InGa QDs, provoke a stronger autophagy flux [97].

### Nanomaterials as blockers of autophagy flux

As mentioned previously, NPs block autophagy flux predominantly due to lysosome impairment resulting from the accumulation of NPs in lysosomes. This can be either due to lysosome overload, altered lysosomal pH and structural impairment in lysosomes. For example, exposure to silica (SiO_2_) NPs impaired the degradative function of lysosomes by increasing lysosome membrane permeabilization (LMP) blocking the autophagy flux on hepatocytes [98]. Similar effects were observed by AuNPs but were dependent on the size. AuNPs of size 50 nm was readily internalized by cells, caused lysosome alkalization eventually resulting in blockade of autophagy flux [99].

Cationic NPs, such as cationic dendrimers (G5) [100], result in the accumulation of protons and subsequently impairment of lysosomes and autophagy [101]. Similarly, Au NPs alkalinize the lysosome and block autophagy through the impairment of its fusion with autophagosomes [102]. Other NPs such as Fe3O_4_ NPs [103], ZnO NPs [104], TiO_2_ [105], CNT [106] and polystyrene NPs [107] are able to block autophagy through an enhance at intracellular ROS level and interfering with autophagosome trafficking resulted from disruption of microtubules and actin polymerization [108]. Autophagy can also block following ER stress. Treatment of macrophages with magnetic iron oxide NP (M-FeNP) caused ER damage and blocked the autophagy flux further leading to increased ER stress [109]. Therefore, to avoid misinterpretation of autophagic effects of NP on cells, it is critical to distinguish between autophagosome accumulation resulting from induction of autophagy flux as opposed to the blockade of autophagic flux [87].

Diving deeper into specific examples, various nanoparticles have shown distinct mechanisms in influencing autophagy flux. For example, silver nanoparticles (AgNPs) have been shown to induce cytotoxicity both in vitro and in vivo by inhibiting the fusion of autophagosome and lysosome [110]. While the use of autophagy inhibitors along with nanoparticles has been an effective approach to treat cancers, Fe2O3@DMSA, a carboxy-functional iron oxide nanoparticle was reported to display anti-tumor effects alone without addition of autophagy inhibitors. The underlying mechanism of inhibitory effect of nanoparticle on hepatoma growth was the blockade of the fusion of autophagosome and lysosome [111]. Moreover, exposure to carbon black nanoparticles during pregnancy exacerbated lung fibrosis induced by bleomycin in offspring via inhibition of autophagy, which was mediated by LKB1-AMPK-ULK1 axis [81]. Excessive accumulation of autophagosomes resulting from blockade in autophagy flux was observed to be mediated by disruption of cytoskeleton in lung cells treated with Graphite carbon nanofibers (GCNF) [112]. Further, decreased mitochondrial activity and membrane integrity was induced by SiNPs in HUVECs with subsequent activation of mitophagy [113]. In addition, exposure of cancer cells to polyethyleneimine (PEI)-coated iron oxide NPs (IONPs) resulted in higher uptake and increased ROS production eventually inducing apoptosis through inhibition of autophagy [114]. Although NMs have shown a beneficial role in autophagy inhibition for the treatment of tumors, understanding how to mitigate damage to normal cells by controlling the level of autophagy induced by these NMs remains a critical area of research. In contrast, the cytotoxic effects and mechanisms of cell death induced by micro- and nanoplastics exposure depend on their size, shape, surface charge, and chemistry, underscoring the complexity of interactions between NMs and biological systems. For instance, polystyrene nPs of size 100 nm were reported to impair autophagic flux in HUVECs [115]. Furthermore, the inhibitory effect of polystyrene nanoplastics (PS-nPs) on autophagic flux was reported to decrease when PS-nPs were interacted with proteins present in fetal bovine serum (FBS). However, the cytotoxic effect of PS-nPs reappeared after the protein corona was degraded in lysosomes. [85]. Figure 2 demonstrates the possible induction or inhibition effect of NMs on the autophagy process.

In primary human nasal epithelial cells polystyrene nPs with size 50 and 500 nm were reported to result in increased expression of autophagy markers, LC3 II and P62 when treated with or without chloroquine (a late-stage inhibitor of autophagy). This is due to the impaired clearance of autophagosomes resulting from impaired autophagic flux. The autophagy marker LC3 II co-localized with fluorescence labelled nPs in cytoplasmic regions [116]. Additionally, mice exposed to 100 mg/kg of PS particles displayed interrupted degradation of autophagic substrates [117]. The interference of accumulated PS particles was confirmed by the colocalization of autophagy markers LC3 II and P62 in mouse embryonic fibroblasts [118]. However, the potential of secondary microplastics to inhibit autophagy has not been extensively studied. Furthermore, research on the impact of secondary microplastics on human and environmental health is still insufficient. Nevertheless, primary microplastics with definite shape and size have been well explored for their impact on human health the understanding of which is important to study the toxic effects of secondary microplastics. Effects of nanoparticles on modulation of autophagy are listed in Table 1.

**Table 1:** NPs, NMs and nPs mediated modulation of autophagy with their underlying mechanism

SNo	Nanoparticle	Model	Effect on autophagy	Mechanism
I. NMs, NPs and nPs as activators of autophagy and inducers of autophagic cell death				
1	Carboxy-functional iron oxide nanoparticle	SK-Hep-1 and HepG2, and HL-7702	Induction	ROS generation [111]
2	AgNPs	HT-29 colon cancer cells	Induction of late non-canonical autophagy	Upregulation of LC3-II [119]
3	AgNPs	SH-SY5Y cells	Induction of Protective autophagy	Ca2 + /CaMKKβ/AMPK/mTOR [120]
4	AgNPs	HeLa	Activation of pro-survival autophagy	PtdIns3K and mTOR dependent [121]
5	AgNPs	HOS CRL-1543 and Huh7	Activation of pro-survival autophagy	ROS generation [122]
6	Copper Oxide Nanoparticles (CuO NPs)	A549 cells	Autophagic cell death	Upregulation of LC3-II [123]
7	Selenium nanoparticles	HaCaT, human keratinocytes	Autophagic mediated cell death	AMPK dependent pathway [124]
8	Gold Nanoparticle	MDA-MB-231, SUM-1315, MDA-MB-468, and HCC1937 TNBC cells	Induction of apoptosis supported by autophagy	Upregulation of autophagy markers along with caspases [125]
9	(rGO–Ag-NPs) reduced graphene oxide–silver nanoparticle nanocomposite	HeLa	Stimulation of autophagy mediated cell death	ROS generation and autophagosome accumulation [45]
10	Zinc Oxide nanoparticles	Primary astrocytes	Induction of autophagy and apoptosis	Activation of PI3K/MAPK pathway [126]
11	Titanium dioxide nanoparticles	RAW 264.7 cells	Activation of autophagy	Increased expression levels of autophagy-related proteins LC3 and Beclin-1 [127]
12	Titanium Dioxide Nanoparticles Nitrogen-doped titanium dioxide (N-TiO_2_) NPs N-TiO_2_ NPs	Primary human keratinocytes A375. Human melanoma cells K562 cells	Activation of cytoprotective autophagy Photoactivation mediated blockade of autophagy flux ROS generation	Upregulation of autophagy markers LC3-II [128] Pleiotropic effect on autophagy [129] Induction of autophagy [96]
13	Platinum nanoparticles	Extravillous trophoblast (EVT) cell lines	Activation of autophagy	Increased conversion of LC3-I to LC3-II and decreased P62 levels [130]
14	CD-Ce6-3BP NPs	4T1 mice model of tumour metastasis	Induction of autophagy induced cell death	ROS and starvation dependent activation [131]
15	Iron oxide NPs	Lung epithelial cancer cells	Autophagy induction	ROS dependent[132]
16	PLT@BPQDs-HED NPs (black phosphorus quantum dots)	RAW264.7 and MCF-7	Induction of autophagy and apoptosis	Upregulation of autophagy and pro-apoptotic factors [133]
17	Iron oxide nanoparticle	HepG2, Huh 7 and Alexander hepatoma cell line, PLC/PRF/5	Induction of autophagic flux	Bcl-2 and p53-mTOR Axis Signaling [134]
18	Ceria-Zirconia antioxidant nanoparticles (PEG-CZNPs)	human renal proximal tubular epithelial cells (HK-2) and human podocytes	Enhancing autophagic flux	Increase in expression levels of autophagy markers [135]
19	Iron (III)-Tannic Molecular Nanoparticles	HepG2.2.15 cells and AML12 cells	Induced autophagic cell death	Upregulation of autophagy markers [136]
20	Copper Oxide Nanoparticles	MCF-7	Induced autophagic cell death	Activation of excess autophagy [137]
21	Polystyrene nanoparticles	HeLa	Activation of autophagy	Activation of TFEB [138]
22	Polystyrene nPs	H9C2 cells	Induction of autophagy	ROS/TGF-β1/Smad [139]
23	Polystyrene nPs	MEFs	Induction of autophagy	Oxidative and inflammatory stress [118]
24	Polystyrene microplastics	GC-2 cells	Activation of autophagy	PINK1/Parkin pathway [140]
25	Polystyrene microplastics	C57BL/6 mice model	Enhanced autophagic activity	Increased levels of ATG5, Beclin-1, and ATG7 [141]
26	Nanosized copper particles	The rat mesangial cell line (HBYZ-1)	Activation of autophagy	Elevation of autophagy markers [142]
II. NMs, NPs and nPs as inhibitors of autophagy and blockers of autophagy flux				
27	Palladium nanoparticles	HeLa	Induction of autophagy and inhibition of autophagy flux	mTOR signalling pathway and lysosome impairment [143]
28	Silica Nanoparticles	HepG2 cells, hepatocytes	Autophagy dysfunction	Lysosome impairment and downregulation of enzymes in lysosomal lumen [98]
29	Nuclear-targeted nanoparticles	bEnd.3 cells	Inhibition of autophagy	Inhibition of PI3K/Akt/mTOR pathway [144]
30	AgNPs	THP-1 monocytes	Blockade of autophagic flux	Impairment of autophagosome and lysosome fusion [145]
31	Gold nanoparticles	HeLa	Impairment of autophagic flux	Lysosomal swelling and impairment [146]
32	Silver nanoparticle	Male ICR mice and BV2 cells	Impairment of autophagic flux	Alterations in the lysosomal acidic environment[147]
33	Graphene oxide nanoparticles	F98 cells	Interrupted autophagic flux	Down regulation of PI3K/Akt/mTOR and impairment of lysosome function[148]
34	Silica nanoparticles	A549 and BEAS-2B	Blockage in autophagic flux and apoptosis	Upregulation of pro-apoptotic factors and autophagy markers[149]
35	Silver nanoparticles	NCIH292 cells, BEAS-2B cells and primary rat type-II cells	Block autophagic flux	Modulation of RIG-I-IRF-7 pathway [150]
36	Gold nanoparticles	Normal rat kidney (NRK) cells	Blockade of autophagy flux	Alkalinization of lysosomal pH and lysosome impairment [99]
37	Titanium dioxide nanoparticles	HeLa	Blockage in autophagic flux	Impairment of lysosomal function [151]
38	Solid Lipid Nanoparticles	PC12 Cells	Suppression of autophagy	Decrease in the expression of autophagy markers [152]
39	Polystyrene nanoplastics (PSnPs)	Intestinal epithelial cells	Impaired autophagic flux	Accumulation of PSnPs [153]
40	Polystyrene nanoplastics (PSNPLs)	Primary Human Nasal Epithelial Cells	Defective autophagy	accumulation of LC3-II and p62 [116]
41	Amine-modified polystyrene nanoparticles	1321N1 cells	Deregulated autophagy	Increase in lysosomal membrane permeability and lysosome dysfunction [80]
42	Polystyrene nanoplastics (PS)	Mice enterocytes	Defective autophagy	Lysosome dysfunction and accumulation of autophagic substrates [117]

### Nanostructures as carriers for autophagy-modulating drugs

In many drug-delivery studies, biocompatible NMs have been developed and optimized as an approach to enhance the active or passive targeting, cellular uptake, systemic circulation of the nanocarriers, and in general the anticancer efficacy of anti-cancer drugs, including Rapamycin, Everolimus, and Dactolisib [154–156]. The same logic goes for the nanomaterial-mediated delivery of autophagy modulating drugs which may target different key elements in the process of autophagy including the mTOR kinase and autolysosomes. In fact, autophagy modulator drugs are currently considered as a promising approach to treat cancer in combination therapies [157]. For example, a thermo-responsive nanocomposite gel provided the vehicle for sustainable drug delivery of two autophagy promoting drugs, paclitaxel and temozolomide, and resulted in synergistic antiproliferative autophagy induction both in vitro and in vivo [158]. The encapsulation of 3-methyladenine (3-MA), a well-known PI3K inhibitor in nano-sized zeolitic imidazolate framework crystals has also been studied [159]. These metal–organic framework nanoparticles were proved to be efficient drug delivery vehicles that enhanced the cellular uptake and blockade of autophagosome formation in HeLa cells. Another compound with autophagy modulating properties is the anti-malaria and anti-rheumatoid drug, chloroquine, which has been used in clinical trials for solid tumours [160]. This FDA approved autophagy blocker is a lysosomal lumen alkalizers that mediates lysosomal dysfunction, impaired degradation of the cargo, and therefore induces block of autophagic flux [161]. A multifunctional delivery system was designed for a pH-responsive targeted delivery for chloroquine diphosphate, a chloroquine derivative as an autophagy blocker [162]. The zinc-based metal–organic framework body was presented as a carrier encapsulating the anti-cancer drug, while folic acid and polyethylene glycol (PEG) coated on the surface worked as targeting and stability improving agents. Nanostructure-based strategies are for multifunctional and synergist approach in cancer therapy, however, the pharmacokinetics, biodistribution, and toxicological patterns of such innovative tools should be taken into account before reaching final clinical stages [163].

### Autophagy-modulating nanomaterials in clinical practice

Autophagy is a process that is involved in a variety of diseases, and researchers have been working to translate findings from the lab to clinical practice. As described previously, the use of NMs carrying drugs or other compounds with the potential to modulate autophagy has been widely explored as therapeutic approach in in vitro and in vivo models. In addition, several nanomedicines carrying drugs inducing autophagy [164–169] have been evaluated in clinical trials, including MM-398 (Irinotecan liposome injection), CRLX101 (Ceramide nanoliposome injection), MBG453 (Anti-TIM-3 antibody nanobody), Cytodrox (Nano Doxorubicin), Abraxane (NanoAlbumin-bound Paclitaxel), and SC-01 (Nanoliposomal C6-ceramide).

**Fig. 3:**
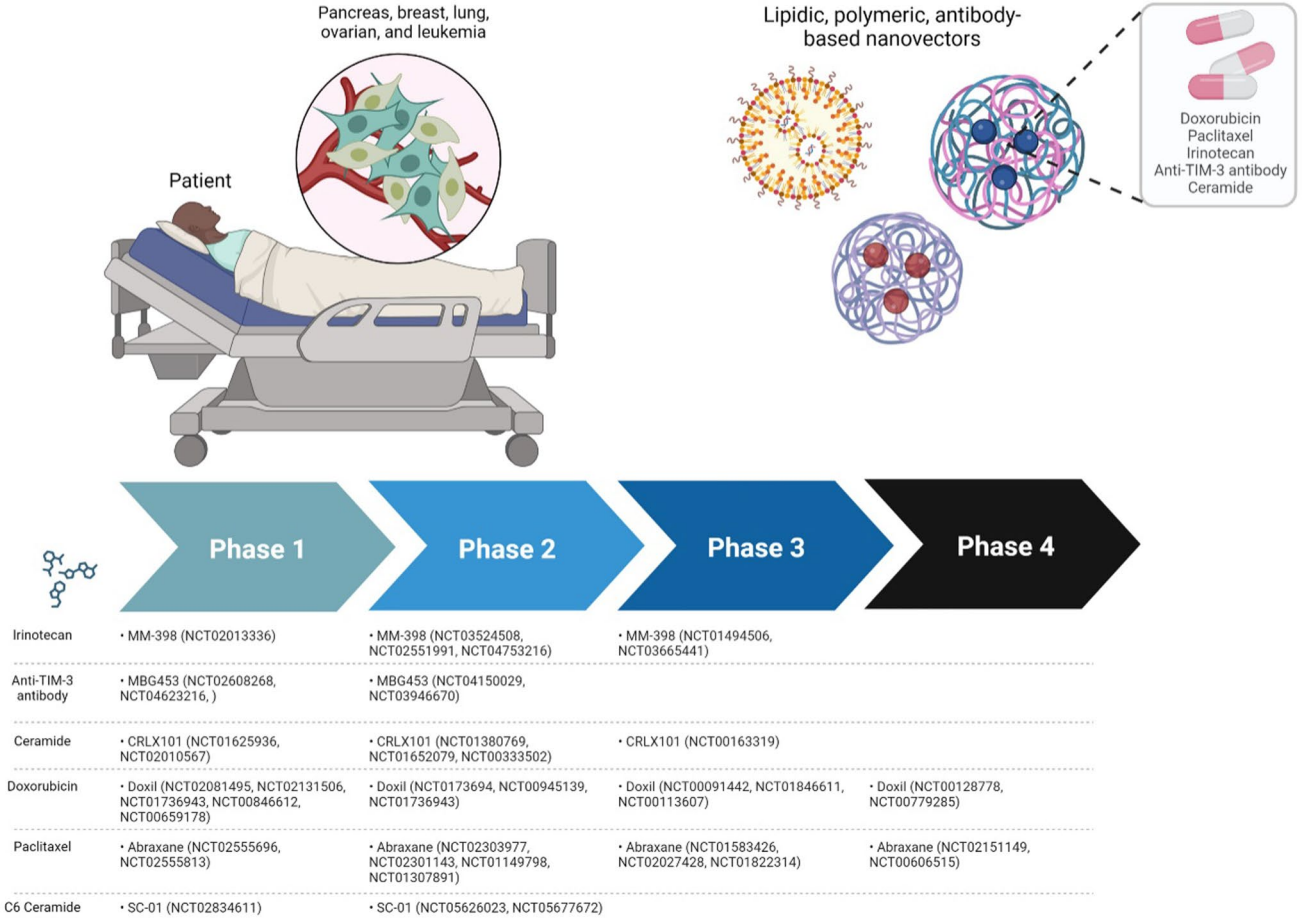
Approved trials with NMs as drug carriers. NMs carrying anticancer drugs based on autophagy modulation have been employed for the treatment of cancer. Here, examples of approved trials involving bioactive molecules at different clinical phases are reported. This figure was created using Biorender.com

A phase I clinical trial evaluated MM-398 in patients with advanced solid tumors, and the results showed that the drug had a favorable safety profile and antitumor activity [170]. In a phase III clinical trial (NAPOLI-1), MM-398 in combination with 5-fluorouracil and leucovorin significantly improved overall survival in patients with metastatic pancreatic cancer who had previously received gemcitabine-based therapy [171]. A phase II clinical trial evaluated CRLX101 in patients with advanced non-small cell lung cancer, and the results showed that the drug had a favorable safety profile and antitumor activity [172]. In a phase I clinical trial, MBG453 demonstrated antitumor activity and a favorable safety profile in patients with advanced solid tumors [173]. A phase II clinical trial is ongoing to evaluate the safety and efficacy of MBG453 in combination with a PD-1 inhibitor. Cytodrox has been evaluated in several clinical trials for the treatment of various types of cancer, including breast cancer, ovarian cancer, and leukemia [174]. Abraxane has been approved for the treatment of several types of cancer and has been designed to enhance drug delivery and reduce toxicity compared to the free drug [175–177]**.** SC-01 has been evaluated in a phase I clinical trial in patients with advanced solid tumors, and the results showed that the drug had a favorable safety profile and demonstrated preliminary antitumor activity [178]. SC-01 is being developed by Spherium Biomed. Other examples of approved clinical trials involving drugs modulating autophagy and nanoparticle are reported in Fig. 3.

In the context of cancer treatment, the dual role of nanomaterial-induced autophagy is highlighted by the work of Shi et al. [90]. They developed a biomimetic nanoformulation that co-encapsulates Oxaliplatin (OXA)/hydroxychloroquine (HCQ) a well-known autophagy inhibitor. The nanoformulation specifically inhibits autophagy, reducing tumor cell migration in vitro and decreasing tumor metastasis in vivo, making it a promising strategy for clinical therapy of hepatocellular carcinoma [91].

This wealth of clinical trial data underscores the transformative potential of autophagy-modulating NMs in clinical practice, opening new horizons for innovative and effective treatments in the battle against various diseases, particularly cancer.

## Nanomaterials induce pro-survival or pro-death autophagy?

NMs can be considered a unique class of autophagy modulators, and in many cases, NMs-induced autophagy promotes cell death [179]. Impaired autophagy can be an indicator of pathogenesis of diseases [180, 181]. Generally, cells treat NPs as particulate pathogens and make an attempt to degrade them. It is challenging to determinate the cell fate based on activation of autophagy that occurs at the initial stage post uptake of NPs. However, NPs are engulfed by autophagosomes [130, 182], which is influenced by factors such as the size and concentration of NPs entering the cell.

Importantly, different concentrations of a same NP may induce both pr-survival and pro-death pathways. For instance, an autophagy-dependent differentiation or autophagy-associated cell death is triggered by low (10 μg/mL) and high (100 μg/mL) concentrations of photoactivated N-TiO_2_ NPs in leukemia K562 cells [96]. Mitophagy, the degradation of damaged mitochondria through autophagy) has been reported as a cytoprotective mechanism against CuO NP-induced cytotoxicity [183].

The pro-death roles of autophagy may be related to the direct induction of autophagic cell death or autophagy may contribute to the induction of other regulated cell death (RCD) pathways, such as apoptosis, necroptosis, ferroptosis and cuproptosis [87, 184, 185]. Indeed, NPs may potentially trigger different forms of RCD [186]. For instance, CuO and TiO2 NP NPs activate ERK-dependent autophagic cell death through triggering ROS [187], while other reports suggest that cytotoxic mechanisms of CuO NPs may resulted from a crosstalk between apoptosis and autophagy [96, 188].—Such cell death mechanisms, however, can be targeted in therapy for apoptosis-resistant cells where cell death is restored upon triggering the autophagy-dependent apoptosis pathway [189, 190].Therapying this content, a natural nanocarrier apoferritin encapsulated in a Cu(II) complex showed cell death autophagy-dependent apoptosis as a sign of cytotoxicity toward various drug resistant cancer cell lines [191]. Other less conventional pathways of RCD can be triggered along with autophagic flux. For example, Gupta et al. showed that induction of mitophagy as well as oxidative and proteotoxic stresses by CuO NPs led to the induction of cuproptosis, a form of RCD that triggered by the accumulation of Cu in mitochondria [192].In addition, iron oxide NPs can be inducers of autophagy-dependent ferroptosis, a form of RCD that is driven by iron-dependent phospholipid peroxidation through the activation of autophagy machinery [193]. In this case, ultrasmall iron oxide (USIO) NPs have been applied for glioblastoma cells to induce ferroptosis via a Beclin1/ATG5-dependent autophagy pathway by increasing the intracellular iron level, catalysing Fenton reaction, generating ROS and lipid peroxidation [194].

PAMAM nanoparticles promoted autophagy mediated cell death causing acute lung injury via Akt-TSC2-mTOR signaling [195]. Similarly, autophagy mediated by PAMAM dendrimers was reported to cause neuronal cell death [196]. In addition to endo-lysosomal pathways, NMs are also engulfed within autophagosomes and are degraded through auto-lysosomal pathway [197]. Autophagy mediated by iron oxide nanoparticles is often pro-death [109], and silica nanoparticles disrupted endothelial cell homeostasis, leading to impaired angiogenesis by elevating autophagic activity [198]. Excess autophagy was reported to be the underlying mechanism in MWCNTs induced neurotoxicity [199]. Many NMs have the potential to be used as anticancer agents and the cytotoxic effects exerted by NMs towards cancer cells remains the treatment strategy for cancers. Furthermore, modulation of autophagy using autophagy modulators along with NMs can facilitate optimization of killing of cancer cells.

NMs inducing pro-death autophagy can be used as nanomedicines for cancer therapy [200]. As discussed above, cellular events like ROS generation, oxidative stress, mitochondrial damage, and even lysosomal impairment precede nanomaterial induced cytotoxicity which can be exploited for treatment of cancers. Similarly, autophagy plays a protective role against toxic impact of NPs. Activation of autophagy decreased the levels of pro-inflammatory cytokines, TNF-α and IL-1β secreted during dextran-coated Fe_3_O_4_ NP induced inflammation [201]. In addition, autophagy triggered by both MSNs [202] and curcumin-loaded selenium NPs [203] diminished NF-κB mediated inflammation. Cells activated autophagy to mitigate Endoplasmic Reticulum (ER) stress induced by SiO_2_ NPs [204]. Recently, nanoparticles have been largely explored for wound healing application [60]. Autophagy is the key mechanism involved in the process of wound healing [205] 205. However, the use of nanoparticles to induce wound healing with autophagy as a direct or indirect mediating mechanism has not been widely reported and remains a gap in therapeutic interventions driven by nanotechnology in wound healing. While NMs that induce pro-death autophagy can be potential targets for cancer therapy, NMs that induce pro-survival autophagy can be considered for wound healing, antioxidant formulations for dermatological applications. AuNPs, nearly spherical in shape, of size around 36 nm, stabilized with serum proteins from fetal bovine serum, induced formation and accumulation of autophagosomes accompanied by upregulated stress-response proteins and antioxidants in MRC-5 cells [207].

Cerium oxide NPs promoted the clearance of proteolipid aggregates in fibroblasts derived from infantile neuronal ceroid lipofuscinosis patients by inducing autophagy-mediated activation of transcription factor EB [208]. In Neuro 2A cells, autophagy induced by europium hydroxide nanorods (EHNs) facilitated the degradation and clearance of mutant huntingtin protein aggregation via MEK/ERK1/2 signaling pathway [209]. Furthermore, Au NPs of size 45 nm were reported to induce osteogenesis through the activation of autophagy. As the inhibition of autophagy with 3-Methyladenine, a well-known autophagy inhibitor, reversed the angiogenesis process, active autophagy was confirmed to be the underlying mechanism [210]. In addition to the protective role of NPs evoked autophagy, simultaneous impairment in the autophagy flux is a rising concern. This is predominantly due to the accumulation of NPs, impaired clearance mechanisms. For example, Palladium NPs induced autophagy in HeLa cells and also resulted in the inhibition of autophagy flux [143].

To further exemplify the therapeutic potential of modulating autophagy with NMs, known autophagy inhibitors can be used to inhibit pro-survival autophagy induced by NMs to kill cancer cells. Zhang et al., demonstrated this by using Ag NPs to induce autophagy and increasing autophagosomes without disrupting lysosome function and cargo degradation. Interestingly, they reported that inhibition of autophagy using wortmannin increased Ag NPs induced cell death in mouse B16 melanoma model indicating that Ag NPs induced pro-survival autophagy [121].

Similarly, pro-survival autophagy can be modulated to develop and improve the anti-cancer efficacy of NMs [211]. Additionally, cellular ROS production post uptake of NPs precedes autophagy activation [81, 122, 212]. Therefore, NPs that can augment cellular ROS levels can trigger autophagy. However, the type of autophagic response towards cell death and survival varies depending on physicochemical properties of NPs. On the other hand, disrupted autophagy or blockade at different stages of the autophagy pathway can lead to pathogenesis of diseases and often leads to cell death. The disruption may result from accumulation of NPs within autophagosomes, impairment of lysosomal stability eventually decreasing autophagic flux by inhibiting the fusion of autophagosomes to lysosomes. This results in the accumulation of damaged proteins, DNA and organelles increasing the risk of neurodegenerative diseases [91] and cancer [87]. NMs can override the pro-death nature of autophagy by stimulating and upregulating pro-survival factors. Additionally, it is also possible that the cell debris accumulated from NPs induced cell death (via apoptosis or necrosis) requires the activation of autophagy for clearance. However, this process is often misinterpreted as autophagic cell death. In such cases, the use of autophagy inhibitors does not inhibit cell death. Therefore, autophagy cannot be confirmed as a killing event but a bona fide process of cell death [213].

**Fig. 4:**
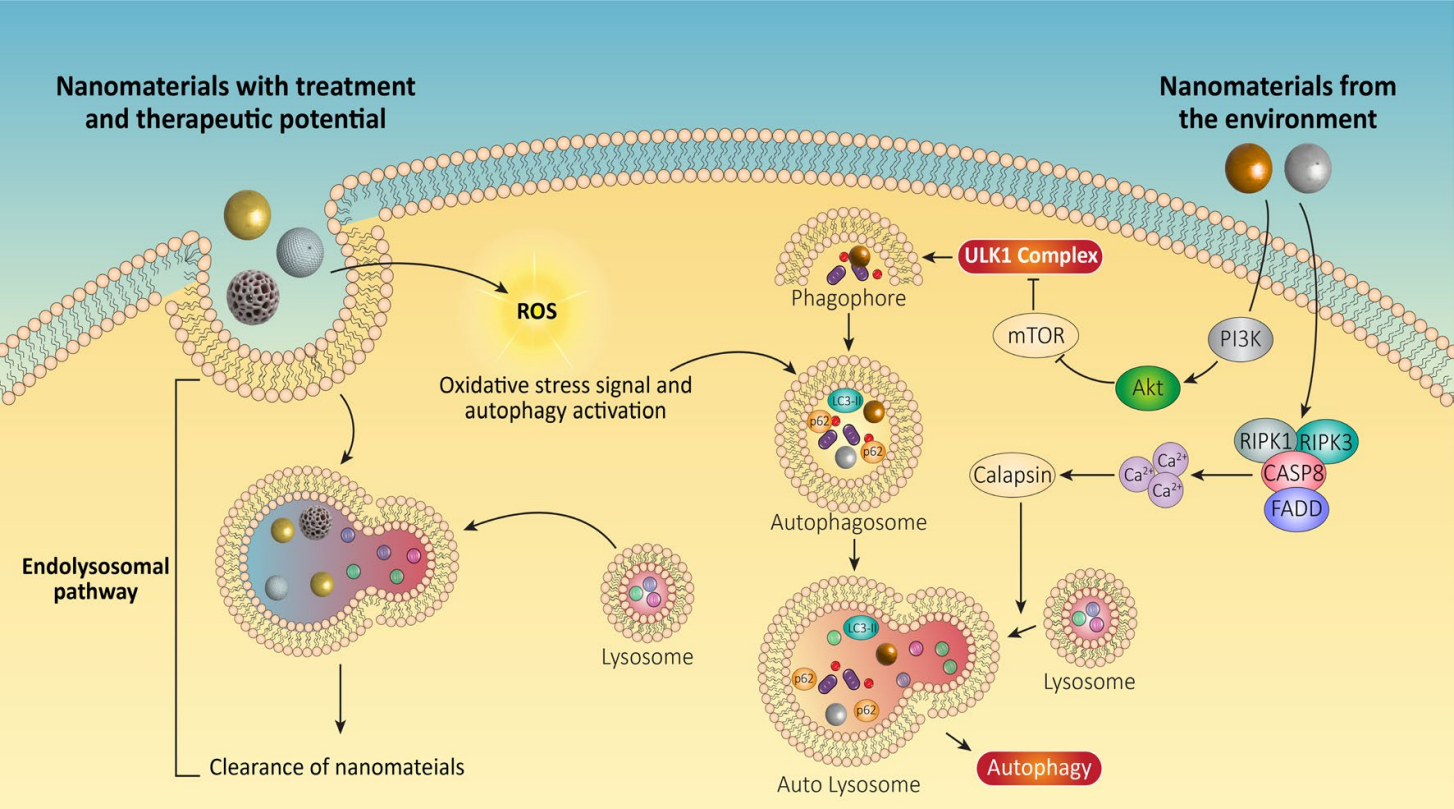
NMs differentially regulate autophagy. Schematic representation of autophagy being a pro-death and pro-survival mechanism in response to nanomaterial exposure is showed here. Autophagy is also activated as a supporting mechanism for cell death

On the other hand, autophagy remains one of the major cellular responses against exposure to micro and nanoplastic particles. The toxicity induced by nPs was attenuated by canidin-3 glucoside via activation of autophagy [214]. Internalization of 100 nm of polystyrene nPs caused damage to cell membrane in HUVECs triggering autophagosome formation and indicating the initiation of autophagy. However, PS-NPs caused impairment in autophagy flux [115]. Exposure to PSNPs/LPS disrupted myocardial structure and exacerbated autophagy and myocardial fibrosis in mice through ROS generation [139]. Furthermore, PSNPs impaired autophagic flux in intestinal epithelial cells affecting their survival and growth [153]. Interestingly, several studies have reported the non-cytotoxicity of PSNPs both in vitro and in vivo. However, despite being non-cytotoxic, PSNPs were reported to have altered several cellular responses [215]. These reports suggest that NMs inducing autophagy, both pro-death and pro-survival, can be promising targets for use as nanomedicine for treatment of several diseases. Schematic representation of pro-death and pro survival autophagic response to NMs is shown in Fig. 4.

## Autophagy: indicator for nanomaterial toxicity

The activation of autophagy by nPs can indicate either the toxic impact of NMs to induce cytotoxicity via autophagic cell death or the cell’s attempt to alleviate nPs induced stress and toxicity. Similarly, nPs mediated inhibition of autophagy flux mainly represents their toxicity [216]. However, this is different in the case of nanoparticles intended for treatment and therapeutic strategy as many NMs are synthesized and designed to target and modulate autophagy. Additionally, autophagy activation remains the non-targeted response of cells treated with NMs that are designed to possess therapeutic potential [67]. Although there is no specific pattern of NMs-mediated autophagy modulation to count its induction or inhibition as an indication of nanomaterial’s toxicity, as discussed above, modulation of autophagy remains a major response of cells exposed to NMs, NPs and nPs.

In contrast to other NMs and nano-formulations, cells exposed to nPs majorly display stimulated autophagy [78]. Interestingly, activation of autophagy is an initial response, but autophagosomes tend to accumulate within the cells at later time points due to the accumulation of nPs in lysosomes [215] 215, which impairs autophagy flux. Co-treatment of arsenic (As) and PS-NPs activated excess autophagy eventually inducing apoptosis in mice liver [217]. Remarkably, nPs, particularly polystyrene, induce autophagy that results in cell death [218]. Although the modality of cell death may be different, autophagy activation is one of the preceding responses to nPs exposure. Similarly, stress signals precede autophagy activation. nPs can induce several stress signals that activate autophagy [219, 220]. Activation of autophagy following NMs-mediated stress signals such as oxidative stress and ER stress has been reported [221, 222]. Furthermore, activation of autophagy is a definite response against exposure to nPs across several species. For example, in Saccharomyces Cerevisiae, activation of autophagy as a protective role was also confirmed upon exposure to polyethylene terephthalate nanoplastics (PET-NP) [223]. Nevertheless, whether the autophagy activated in response to nPs is pro-survival or pro-death still needs a clarification. Activation of autophagy as a basic stress response to NM exposure makes the pathway a reliable early detection marker for NM toxicity reinforcing the existing NM toxicity evaluation strategies.

## Challenges and opportunities: the dual role of autophagy in nanoparticles

The intricate relationship between autophagy and nanoparticles (NPs) offers both exciting opportunities and intricate challenges, especially in the realms of clinical and environmental scenarios.

From a clinical perspective, leveraging the power of autophagy in NP therapies can herald groundbreaking treatments. The ability of certain nanoparticles to modulate autophagic processes means they can either boost the cellular defense mechanism or use it as an avenue to introduce therapeutic agents, presenting a unique dual-action mode of therapy [45]. However, challenges arise in ensuring the precise modulation of autophagy for therapeutic purposes. Overstimulating autophagy pathway might lead to unintended cell death, whereas inhibiting it excessively can interfere with the natural defense mechanisms, potentially limiting the very cells we intend to treat [224].

The environmental implications are profound. As our ecosystems face contamination from micro and nanoplastics, understanding how autophagy responds to these foreign entities becomes paramount. Autophagy might serve as a cellular safeguard, attempting to process and neutralize these particles [225]. Yet, autophagy process can be compromised by nPs, affecting cellular health and, on a broader scale, the health of organisms [226]. The silver lining here lies in the potential to engineer NPs that can either enhance autophagy where it is beneficial or inhibit it where it is detrimental, thus offering a means to mitigate environmental risks associated with nPs pollution.

## Conclusion

While nanotechnology driven therapeutic interventions are increasing, almost all the compartments of ecosystem are contaminated with micro and nanoplastics. There is an increased risk of exposure through all routes. Autophagy is currently being explored as a cellular response to nanomaterial exposure and as an exploitable mechanism to facilitate the use of NMs for therapies and treatment of diseases. In this regard, we provided an overview of the clinical potential of autophagy modulation with nano formulations. However, it is also important to consider the role of autophagy as a defensive mechanism against NMs. Autophagy is a fascinating phenomenon in biological systems that bridges the gap between NMs existing in the environment and those intended for use in therapeutics. Nevertheless, further studies are required to understand the effect of nanoscale and submicron plastics on cellular process and deeper organs through circulation. Further, the synthesis and development of more NMs that can effectively modulate autophagy pathway are crucial for future treatment aspects and to set up novel clinical trials.

In conclusion, the interplay between autophagy and NPs holds immense promise, albeit fraught with challenges. The key lies in continued rigorous research, bridging the gap between the therapeutic potential of autophagy-modulating nanoparticles and the environmental imperatives of the present day. It should be mentioned that autophagy should be evaluated carefully for each NP as there is a complex interaction between NPs and the cellular autophagic machinery, which depends on physico-chemical characteristics of NPs, including size, charge dispersity, and concentrations, as well as type and content of cell models.

## Data Availability

Not applicable.
